# PRP IN THE TREATMENT OF TROCHANTERIC SYNDROME: A PILOT STUDY

**DOI:** 10.1590/1413-785220162404159837

**Published:** 2016

**Authors:** Arthur de Góes Ribeiro, Walter Ricioli, Alice Roxo Nobre Sousa e Silva, Giancarlo Cavalli Polesello, Rodrigo Pereira Guimarães

**Affiliations:** 1. Irmandade da Santa Casa de Misericórdia de São Paulo, Department of Orthopedics and Traumatology, São Paulo, SP, Brazil.; 2. Faculdade de Ciências Médicas da Santa Casa de São Paulo, São Paulo, SP, Brazil.

**Keywords:** Bursitis, Femur, Injections, Platelet-rich plasma, Tendinopathy

## Abstract

**Objective:**

To compare the efficacy of platelet rich plasma (PRP) against corticosteroid on the treatment of trochanteric pain syndrome***.***

**Methods:**

From July 2011 to November 2012, eighteen patients (20 hips) with trochanter pain syndrome were randomized in two groups and treated with platelet rich plasma or triamcinolone infiltration guided by ultrasound. Pain and function were evaluated prior to the intervention and after 10, 30 and 60 days, through the Facial Expressions Scale for Pain and the Western Ontario McMaster and Harris Hip Score questionnaires. Inter-group analysis was performed by Student t-test and intragroup analysis by ANOVA, followed by Bonferroni post hoc test. Statistical significance was set at p <0.05***.***

**Results:**

There was no difference between the groups. The triamcinolone group showed pain reduction (p=0.004) and improved function (p=0.036) through the Harris Hip Score questionnaire at 10, 30 and 60 days after treatment, when compared with the pre- intervention period. The platelet rich plasma group showed no statistical improvement in any of the variables***.***

**Conclusion:**

Up to 60 days, PRP infiltration has no influence on pain relief and function improvement in trochanteric syndrome treatment. ***Level of Evidence II, Prospective Comparative Study.***

## INTRODUCTION

The trochanteric pain syndrome (TPS)^1^ ^-^ ^3^ is characterized by chronic pain in the lateral side of the hip.^3^ ^,^ ^4^ It usually occurs unilaterally and in middle-aged women.^3^ The causes are several, such as *coxa saltans* (snapping hip) and trauma, and it is often associated with other musculoskeletal disorders in lower limbs.^2^ The diagnosis is primarily clinical, but further tests they can be used to search for and exclude differential diagnosis.^5^ Many treatments have been described and conservative approach is generally successfull.^5^

The main treatment is done with supportive measures such as physical therapy, analgesics, cryotherapy, weight loss and rest and also by treating diseases such as arthritis, low back pain and lower limbs length discrepancy).^3^ ^,^ ^5^ These measures relieve symptoms in most cases.^5^ When pain is persistent, infiltration with corticosteroids^3^ ^-^ ^5^ may be performed with a success rate of 60-100%.^5^

Platelet-rich plasma (PRP) is an autologous product studied since the late 70s.^6^ It has been used in dentistry, plastic and orthopedic surgery.^6^ ^-^ ^9^ It has a high concentration of platelets and growth factors (VEGF, PDGF, IGF-1, EGF). Platelets are activated and the suspension is applied to the desired location, stimulating vascular proliferation and promoting tissue healing.^10^ ^,^ ^11^ Existing studies in orthopedics show controversial results^9^ ^,^ ^12^ ^-^ ^15^ and most evidences do not provide definitive conclusions about PRP effectiveness.^10^ ^-^ ^17^

To date, there is no consensus on the use of PRP in TPS. The aim of this study is to compare the efficacy of PRP and corticosteroid in pain and function relief in patients with TPS. Our hypothesis is that PRP infiltration promotes better results as compared to corticosteroids.

## METHODS

This is a prospective, randomized, double-blind pilot study. It was approved by the Ethics Research Committee of the Department of Orthopedics and Traumatology of *Irmandade da Santa Casa de Misericordia de Sao Paulo* under number 245/11, registered in the Brazilian Registry of Clinical Trials (RBR-82gqqk) and written according to the standards established by CONSORT. All participants signed a Free and Informed Consent Form.

Between July 2011 and November 2012, patients of both genders aged 18 to 79 years old were selected according to the following criteria: pain in the lateral hip for more than three months, hypersensitivity next to the greater trochanter, pain during forced abduction of the hip and tendinobursitis diagnosed by MRI.^18^ Patients who have had previous infiltration in the hip, joint space narrowing , osteophyte in the hip, spine diseases, neurological diseases associated with motor disorders, fibromyalgia, rheumatic polyarthralgia or any other rheumatological diagnoses were excluded from the study.

Forty-six patients with an initial diagnosis of hip tendinobursitis were selected. Of these, 18 patients (20 hips) met the study's inclusion criteria.

All patients underwent a complete physical examination of the spine and hip (researcher 1), with special attention to painful palpation in the region of the trochanteric bursa and around, range of motion of the hip and investigation for femoroacetabular impact.

All subjects underwent anteroposterior radiographs of the pelvis, Ducroquet profile X-ray of the hip joint, fake Lequesne profile and MRI of the hip. The images were always evaluated by the same radiologist (researcher 2) and were used to search for exclusion criteria and also confirm the diagnosis of trochanteric syndrome.

After inclusion in the study and signing the consent form, subjects were divided into two groups, according to previous randomization (through Sequence Generator http://www.random.org). One group received infiltration with Platelet Rich Plasma (PRP Group, consisting of 9 patients, 10 hips) and the control group (with 10 patients-10 hips) received infiltration with hexacetonide triamcinolone (Triancil^(r)^ 20mg/ml, Apsen, São Paulo, Brazil).

To ensure the blindness of the study, on the day of infiltration, a sample of 25 ml peripheral blood was collected from all individuals from both groups.

**Figure 1:**
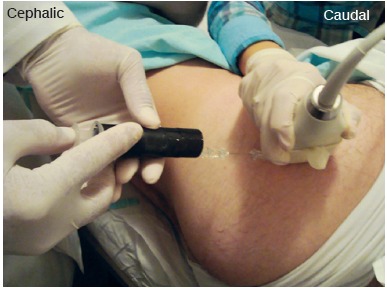
Patient positioning for infiltration. The black tape covered syringe ensures blind infiltration.

The PRP and Triancil^(r)^ solutions were prepared by a biomedical scientist (researcher 3) and applied with a black tape covered syringe to ensure the blindness of the test. (Figure 1)

**Figure 2:**
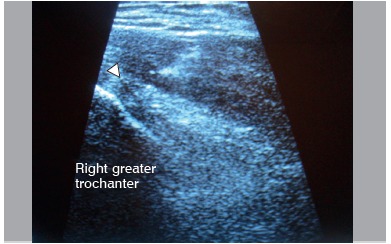
Coronal ultrasound view at the time of infiltration. The arrow indicates the needle at the time of infiltration.

Infiltrations were guided by ultrasound (Philips M2540A, Linear Transducer L12-3, 21475A - São Paulo, Brazil) in order to increase their accuracy, with the aid of the same radiologist.^19^ The patients remained in lateral decubitus on the healthy side and 30 degrees of flexion of the affected hip. The most painful point was identified by palpation and the skin was disinfected with iodine-based solution. Local anesthesia of the skin and subcutaneous tissue was performed with 2 to 5 ml of 2% lidocaine hydrochloride (Xylestesin^(r)^ 20 mg/ml, Cristália, Sao Paulo, Brazil). With a 25G needle, the drug was injected into the trochanteric bursa and around it, according to the size of the affected area (Figure 2). Researcher 1 held all infiltrations.

Individuals were released 40 min later and oriented to apply ice packs on the site every two hours, for the first 24h. They were also instructed not to perform activities that would overload the hip joint for three days.

The use of nonsteroidal anti-inflammatory drugs was not allowed, only Paracetamol 500mg was used, if necessary. Physical therapy was not held in any case and return to sports activity was allowed after 30 days if the patient was asymptomatic.

Patients randomized to the control group received 4ml solution of 80mg of triamcinolone hexacetonide (Triancil^(r))^. The Platelet Rich Plasma (PRP) was prepared by researcher 3. Twenty-five milliliters of peripheral blood from patients were harvested and added to a tube containing 10% citrate phosphate dextrose adenine (an anticoagulant). The blood was centrifuged for 15 minutes at 200g in a table top centrifuge and the fraction containing PRP together with the supernatant (5 ml) was transferred to a second sterile tube. A 1ml aliquot was collected in all cases to determine the final platelet concentration in the sample (Fanem Excelsa II, 206 BL, São Paulo, Brazil). The final volume of PRP preparation was 4 ml, to which 0.1ml of 10% calcium gluconate was added. The mean platelet concentration of PRP was 9.23x10^6^ U/µL.

The primary endpoint was defined as evaluation of pain relief through Facial Expressions Pain Scale (FEPS) prior to intervention and 10, 30 and 60 days after the intervention.

The secondary outcome was defined as function evaluation through the Harris Hip Score questionnaire (HHS) and the Western Ontario McMaster Index (WOMAC) prior to the intervention and 10, 30 and 60 days after the intervention. All evaluations were performed by researcher 1.

A statistical analysis was performed. The normality of the data was checked and the data were expressed as mean and standard deviation. The inter-group analysis was performed using the Student's t-test. The results of the different follow-up times (intragroup analysis) were analyzed using ANOVA test for repeated measures, followed by Bonferroni *post hoc* tests. Statistical significance was set at *p* <0.05. We used SPSS version 18 (SPSS Inc., IBM, Chicago, IL) for all analyzes.

## RESULTS

The mean age of subjects was 49.8 ± 14.6 years old (PRP group, 50 ± 17.8 years old; Control group, 49.6±11.66 years old). Ten subjects (56%) were female (11 hips, PRP group = 3; Control group = 8). Thirteen patients (72%) reported previous treatment and all had pain for more than 6 months. Regarding sports activities, seven subjects (39%) were active despite the discomfort, four (22%) did not practice sports because of pain and 7 (39%) were sedentary. Averages and standard deviations data for each group over the study are shown in Table 1.

**Table 1:** Mean and Standard Deviation, minimum and maximum of the variables FEPS, HHS, and WOMAC from both groups.

	Moment	Corticosteroid	PRP
		Mean ± St. Dev	Mean ± St. Dev
FEPS	Pre	1.9 ± 0.568	3.6 ± 1.17
	10 days	4 ± 1.414	4.4 ± 1.35
	30 days	4 ± 1.247	4.2 ± 1.39
	60 days	4.8 ± 1.549	4.8 ± 1.22
HHS	Pre	57.208 ± 11.5	65.229 ± 12.2
	10 days	78.015 ± 12.7	76.795 ± 16.1
	30 days	75.27 ± 17.3	72.6 ± 14.3
	60 days	79.47 ± 20.4	70.645 ± 14.0
WOMAC	Pre	61.3 ± 20.3	74.1 ± 10.2
	10 days	79.9 ± 18.6	77.2 ± 19.3
	30 days	78 ± 18.5	77.7 ± 10.8
	60 days	81.3 ± 20.2	73.95 ± 14.8

FEPS, Facial Expression Pain Scale; HHS, Harris Hip Score; WOMAC, Western Ontario McMaster; St. Dev., Standard Deviation.

At baseline, 10 hips had normal muscle strength, four hips (20%) had muscle strength grade 4 and six hips (30%) had muscle strength grade 3 (30%) in Kendall scale.

Intergroup analysis showed no statistical difference between PRP and control group after the infiltrations (Table 2) in all analyzed times. When comparing the variables over time, there was a statistically significant difference only for the control group. This group showed reduced pain and improved function according to HHS at 10, 30 and 60 days when compared to the pre-intervention period. (Table 3)

**Table 2:** Intra-group comparison of outcomes*.

	Moment	Mean Difference	Standard error	CI (95%)		*p*
				Inferior	Superior	
FEPS	Pre	1.7	0.41	0.80	2.5	0.001**
	10 days	0.4	0.61	(-)0.8	1.6	0.52
	30 days	0.2	0.59	(-)1.0	1.4	0.74
	60 days	0	0.62	(-)1.3	1.3	1
HHS	Pre	(-)8.0	5.32	(-)3.1	19.2	0.15
	10 days	1.2	6.51	(-)14.9	12.5	0.85
	30 days	2.6	7.10	(-)17.6	12.3	0.71
	60 days	8.8	7.82	(-)25.4	7.7	0.27
WOMAC	Pre	(-)12.8	7.20	(-)2.7	7.2	0.09
	10 days	2.7	8.48	(-)20.5	8.4	0.75
	30 days	0.3	6.79	(-)14.8	6.7	0.96
60 days	7.3	7.93	(-)24.1	7.9	0.36

FEPS, Facial Expression Pain Scale; HHS, Harris Hip Score; WOMAC, Western Ontario McMaster; CI, Confidence Interval 95%; *Control Group - PRP Group. **Statistically significant difference.

**Table 3:** Intra-group comparison of outcomes.*

EEFD						
Groups	Intra-group comparison	Mean Difference	Standard error	CI (95%)		*P*
				Inferior	Superior	
Corticosteroid	Pre - 10 days	-2.1	0.56	-3.66	-0.54	0.004**
	Pre - 30 days	-2.1	0.56	-3.66	-0.54	0.004**
	Pre - 60 days	-2.9	0.56	-4.46	-1.34	0.0001**
	10 days - 30 days	0	0.56	-1.56	1.56	1
	10 days - 60 days	-0.8	0.56	-2.36	0.76	0.97
	30 days - 60 days	-0.8	0.56	-2.36	0.76	0.97
PRP	Pre - 10 days	-0.8	0.57	-2.41	0.81	1
	Pre - 30 days	-0.6	0.57	-2.21	1.01	1
	Pre - 60 days	-1.2	0.57	-2.81	0.41	0.26
	10 days - 30 days	0.2	0.57	-1.41	1.81	1
	10 days - 60 days	-0.4	0.57	-2.01	1.21	1
	30 days - 60 days	-0.6	0.57	-2.21	1.01	1
HHS						
Corticosteroid	Pre - 10 days	-20.8075	7.11	-40.68	-0.94	0.03 **
	Pre - 30 days	-18.0625	7.11	-37.93	1.81	0.09
	Pre - 60 days	-22.2625	7.11	-42.13	-2.39	0.02**
	10 days - 30 days	2.745	7.11	-17.13	22.62	1
	10 days - 60 days	-1.455	7.11	-21.33	18.42	1
	30 days - 60 days	-4.2	7.11	-24.07	15.67	1
PRP	Pre - 10 days	-11.5665	6.37	-29.37	6.24	0.469
	Pre - 30 days	-7.371	6.37	-25.18	10.44	1
	Pre - 60 days	-5.416	6.37	-23.22	12.39	1
	10 days - 30 days	4.1955	6.37	-13.61	22.00	1
	10 days - 60 days	1.955	6.37	-11.66	23.96	1
	30 days - 60 days	1.955	6.37	-15.85	19.76	1
WOMAC						
Corticosteroid	Pre - 10 days	-18.6	8.7	-42.89	5.69	0.23
	Pre - 30 days	-16.7	8.7	-40.99	0.38	7.59
	Pre - 60 days	-20	8.7	-44.29	4.29	0.16
	10 days - 30 days	1.9	8.7	-22.39	26.19	1
	10 days - 60 days	1.4	8.7	-25.69	22.89	1
	30 days - 60 days	-3.3	8.7	-27.59	20.99	1
PRP	Pre - 10 days	-3.1	6.38	-20.93	14.73	1
	Pre - 30 days	-3.6	6.38	-21.43	14.23	1
	Pre - 60 days	0.15	6.38	-17.68	17.98	1
	10 days - 30 days	-0.5	6.38	-18.33	17.33	1
	10 days - 60 days	3.2	6.38	-14.58	21.08	1
30 days - 60 days	3.75	6.38	-14.08	21.58	1

FEPS, Facial Expression Pain Scale; HHS, Harris Hip Score; WOMAC, Western Ontario McMaster. *Control Group - PRP Group. **Statistically significant difference.

We did not observe any adverse effects in this study. There was infection nor muscle-tendon rupture.

## DISCUSSION

This study aimed to compare the efficacy of PRP with that of corticosteroids in pain and function in patients with PTS analyzed before intervention and 10, 30 and 60 days after it. There was no significant difference between the two infiltration techniques, besides not having been observed improvement in pain and function in the PRP group in any of the periods analyzed.

The treatment of TPS is conservative in most cases and may include physiotherapy, analgesia, changing habits and activities of daily living.^5^ The non-respondents to these treatments may be subjected to corticosteroid infiltration,^19^ with 80% satisfactory results, on average of.^5^ ^.^ ^19^ Despite the success obtained after infiltration, there is always risk of tendon degeneration and ruptures.

Experiments with PRP demonstrated an increase in *in vitro* collagen synthesis.^8^ ^,^ ^15^ For this reason, its indication to treat orthopedic injuries^8^ has also increased. A systematic review showed that *in vivo* results of PRP use were highly variable.^16^ Among the reasons for the results variations is the lack of standardization for PRP preparation, which causes difference in the number of platelets and growth factors concentration in the final product.^16^

According to a meta-analysis, only six of 23 randomized clinical studies showed benefits using PRP. A systematic review^11^ included only 16 studies out of 418 due to methodological issues. Of these, only three were considered as reliable. Some studies with PRP have shown benefits in rotator cuff injuries,^9^ in lateral epicondylitis^14^ and chronic patellar tendinitis.^13^ Other studies have shown no advantages by using PRP use in calcaneal tendinitis.^12^ ^,^ ^15^ There was only one reference to the possible use of PRP for TPS treatment,^10^ but no recent publication was found.

It has been shown that several diseases in many body sites have different responses to PRP. Thus, there is no consensus on its use in orthopedics.

In this pilot study, only the control group showed a significant improvement in pain and function (*p*<0.05) using steroids at 10, 30 and 60 days after infiltration, only the HHS questionnaire in 10 and 60 days, as compared to pre-intervention. Still, when comparing the post-intervention periods each other, it was observed that there was no statistically significant difference between them, which shows that these group of patients were not improving over time. There was no significant improvement in function according to HHS at 30 days of follow-up, but we believe that this result was by chance, since there was an improvement after 60 days.

Corticosteroids are used in orthopedic practice for the treatment of articular and extra-articular diseases. Some action mechanisms and pharmacokinetics of deposit steroids are not yet fully understood.

We believe that the stagnation of improvement in pain and function in the control group was due to the fact that the drug infiltrated promotes reduction of the local inflammatory process, therefore, when its effect is over, the patients did not progressively improved.

It is interesting, however, the fact that the improvement presented only in the HHS questionnaire. Historically comparing the questionnaires, we found out that HHS was developed to assess different diseases of the hip submitted to different treatment methods. WOMAC, however, despite being a functional assessment tool used for lower limbs, it was developed for osteoarthritis patients, and this may make it a less specific instrument for the disease presently under study, which may explain our findings.

Another hypothesis is that none of the questionnaires have been created for specific evaluation of extra-articular hip disease. Based on this principle, we indirectly identified HHS as the best among the two available for this task.

To plasma be considered platelet-enriched, it is required its concentration to be higher than 1 x 10^6^ platelets/µL.^20^ Although we have not identified positive results, the average concentration of platelets in PRP in our study was 9.23 x 10^6^ platelets/µL, which is a fair concentration power of the method used and, therefore, potentially beneficial.

This study has some limitations. Firstly, being a pilot study, the small sample size (20 hips). It is known that 90% of patients with TPS have other related conditions, ^1^ which resulted in the exclusion of a large number of subjects. Anyway, although we found a statistically significant difference, we believe that our results should be analyzed with caution.

Secondly, the concentration of growth factors has not been measured; such information could have contributed to a better understanding of the action of PRP. Finally, the patients in this study were not submitted to physical therapy. We chose to not indicate physical therapy after infiltration so that any possible improvement found could be attributed to the action of the applied medicines, eliminating any bias.

## CONCLUSION

There was no difference in pain and function between treatment of TPS with infiltration of PRP and corticosteroids. Only the control group had reduced pain and function according to HHS at 10, 30 and 60 days, as compared to pre-intervention period. There was no improvement in the PRP group during follow-up.

More controlled studies with longer follow-up should be conducted to better understand the indications and benefits of PRP in orthopedic diseases.
